# Environmental factors, winter respiratory infections and the seasonal variation in heart failure admissions

**DOI:** 10.1038/s41598-021-90790-7

**Published:** 2021-05-28

**Authors:** Doron Aronson

**Affiliations:** 1 Department of Cardiology, Rambam Medical Center, POB 9602, 31096 Haifa, Israel; 2 B. Rappaport Faculty of Medicine, Technion Medical School, Haifa, Israel

**Keywords:** Cardiology, Risk factors

## Abstract

Seasonal cycles of AHF are causally attributed to the seasonal pattern of respiratory tract infections. However, this assumption has never been formally validated. We aimed to determine whether the increase in winter admissions for acute heart failure (AHF) can be explained by seasonal infectious diseases. We studied 12,147 patients admitted for AHF over a period of 11 years (2005–2015). Detailed virology and bacteriology data were collected on each patient. Meteorological information including daily temperature and relative humidity was obtained for the same period. The peak-to-low ratio, indicating the intensity of seasonality, was calculated using negative binomial regression-derived incidence rate ratios (IRR). AHF admissions occurred with a striking annual periodicity, peaking in winter (December-February) and were lowest in summer (June–August), with a seasonal amplitude (January vs. August) of 2.00 ([95% CI 1.79–2.24]. Occurrence of confirmed influenza infections was low (1.59%). Clinical diagnoses of respiratory infections, confirmed influenza infections, and influenza-like infections also followed a strong seasonal pattern (*P* < 0.0001; Peak/low ratio 2.42 [95% CI 1.394–3.03]). However, after exclusion of all respiratory infections, the seasonal variation in AHF remained robust (Peak/low ratio January vs. August, 1.81 [95% CI 1.60–2.05]; *P* < 0.0001). There was a strong inverse association between AHF admissions and average monthly temperature (IRR 0.95 per 1℃ increase; 95% CI 0.94 to 0.96). In conclusion, these is a dominant seasonal modulation of AHF admissions which is only partly explained by the incidence of winter respiratory infections. Environmental factors modify the susceptibility of heart failure patients to decompensation.

## Introduction

The natural history of heart failure is characterized by acute decompensation episodes, which are the leading cause for hospitalization among patients older than 65 years of age, with over 1 million annual primary admissions^1,2^. Increasing signs and symptoms of congestion are the main reasons why patients with acute heart failure seek urgent medical care^3^. Although some patients are admitted with a clear correctable trigger, a clear precipitating factor cannot be identified in many patients.

Some cardiovascular diseases, including heart failure, follow a seasonal variation pattern of exacerbation^4,5^. Previous studies showed a rise in acute heart failure admissions in the winter^4,5^. This pattern was described both in the northern^6^ and the southern hemispheres^7^.

The underlying mechanism for this phenomenon and which patients are more prone to suffer acute exacerbation is not known, but it is frequently assumed that the increase in AHF admission may follow the seasonal pattern of infectious diseases, particularly respiratory tract infections^5^. However, some previous studies relied on administrative databases based on discharge diagnoses, included both acute and chronic heart failure patients, and lacked clinical data other than age and gender^4,5^. None of these studies formally investigated the extent to which seasonal variation is explained by specific climatic factors and the potential causal relationship between infections and excess winter admissions.

In the present study, our objectives were to determine whether the increase in winter admissions for AHF can be explained by specific patient’s characteristic or triggering by seasonal infectious diseases.

## Methods

We use a database of patients admitted to the Rambam Medical Center, Haifa, Israel with the primary diagnosis of AHF during the period of January 2005 and December 2015. Eligible patients were those hospitalized as with new-onset or worsening pre-existing or new heart failure as primary cause of admission^8^. The study was initiated after receiving approval from the Rambam Health Care Campus Institutional Review Board and Ethics Committee on human research (Approval ID: RMB-15–0539). The need for a written informed consent was specifically waived by the Rambam Health Care Campus Institutional Review Board and Ethics Committee on human research because of the retrospective nature of the study. The study was conducted in accordance with the Ethical Guidelines for Biomedical Research on Human Subjects (Israel Ministry of Health) and in accordance with the principles of the Declaration of Helsinki.

### Meteorological data

We collected the meteorological information including daily temperature and relative humidity (measured at 8 time points each day: 2:00, 5:00, 8:00, 11:00, 14:00, 17:00, 20:00 and 23:00), for the same period from 4 weather monitoring stations in the Haifa area, using data from the Israeli Meteorological Service. The raw daily temperature and humidity data were transformed into monthly averages to provide a single monthly measure of temperature and humidity.

### Measures of influenza incidence and bacterial infections

For each patient we obtained all viral and bacterial tests performed during hospital stay. Fever was defined as core temperature of > 38.0 °C (100.4°F)^9,10^. Cases with concomitant influenza were identified by PCR assays of nasopharyngeal samples. Influenza-like illness (ILI) was defined as fever (temperature of 38 °C or greater) with compatible clinical presentation (cough and/or sore throat) leading to a negative specimens for respiratory viruses^11^. The ILI syndrome can develop in with a wide range of viral agents, including influenza, respiratory syncytial virus, rhinovirus, coronavirus, adenovirus, and parainfluenza. Patients treated with Oseltamivir were considered as having ILI if viral specimens were negative. Clinical diagnoses of pneumonia, acute upper respiratory infection, and acute bronchitis were also collected.

### Statistical analyses

The baseline characteristics of the groups were compared using ANOVA for continuous variables and by the χ2 statistic for categorical variables. Continuous variables without a normal distribution are presented as median (interquartile range [IQR]) and were compared using the Kruskal–Wallis test.

The statistical analysis of seasonal variations was based on Edwards’ approach, or the Walter–Elwood test (using exact month lengths)^12^. The peak-to-low ratio, indicating the intensity of seasonality (equivalent to a risk ratio that contrasts peak month versus the trough month risks for AHF admissions) was calculated based on the method of Brookhart and Rothman^13^, or by calculating incidence rete ratios.

To estimate temporal trends in the occurrence of AHF admissions based on monthly counts, we fitted a truncated negative binomial regression models, with calendar month as the main predictor, and calendar year as an adjusting variable. The outcome was monthly aggregate cases (adjusted for month length), with resulting coefficients as incidence rate ratios (IRR) with reference to August^14^. Similar models were fit to assess the effect of season (defined a-priori as December to February: winter, March to May: spring, June to August: summer, September to November: autumn), mean monthly temperature, and mean monthly humidity. The amplitude of the seasonal effect was was calculated as the peak-to-low ratio, interpreted as a measure of IRR that compares the month with the highest incidence (peak) with the month with the lowest incidence (low or trough)^14^. To compare the seasonality patterns with and without respiratory infections, analyses were repeated after excluding patients with clinical or laboratory evidence of respiratory infections.

Differences were considered statistically significant at the 2-sided *P* < 0.05 level. Statistical analyses were performed using the Stata version 16.0 (College station, TX).

## Results

We used a database of all AHF admissions during the period of January 2005 and December 2015 including 12,147 admissions. Table 1 displays the clinical characteristics of the study cohort according to the season of admissions. Differences in clinical characteristics were small, albeit significant for some.

**Table 1:** Baseline patient characteristics according to time-of-year at admission.

Characteristic	Season of year				*P* value
	Dec–Feb (n = 3,844)	Mar–May (n = 3,388)	Jun–Aug (n = 2274)	Sep–Nov (n = 2641)	
Age (years)	75 ± 13	74 ± 14	73 ± 15	73 ± 15	0.0002
Female gender	1926 (50)	1617 (48)	1133 (50)	1301 (49)	0.21
BMI	29.3 ± 6.1	29.5 ± 6.2	29.1 ± 6.1	29.4 ± 6.2	0.77
Hypertension	2421 (63)	2066 (61)	1329 (58)	1652 (63)	0.003
Diabetes mellitus	2034 (53)	1806 (53)	1119 (49)	1341 (51)	0.007
Chronic lung disease	500 (13)	474 (14)	335 (15)	358 (14)	0.27
Atrial fibrillation	1591 (41)	1440 (43)	944 (44)	1108 (42)	0.75
Systolic blood pressure (mm Hg)	142 [123–168]	139 [121–163]	136 [118–159]	140 [120–163]	< 0.0001
Baseline creatinine (mg/dL)	1.1 [1.0–1.9]	1.4 [1.0–1.9]	1.3 [1.0–1.9]	1.3 [1.0–1.9]	0.42
Baseline eGFR (ml·min^**−**1^/1.73 m^−2^)	44 [29–63]	44 [29–63]	44 [28–64]	43 [28–63]	0.73
Baseline BUN (mg/dl)	28 [20–44]	29 [20–45]	29 [20–44]	28 [20–44]	0.15
Serum sodium (mmol/l)	137 ± 5	137 ± 5	137 ± 5	137 ± 4	0.02
Baseline haematocrit (%)	35 ± 6	35 ± 6	35 ± 6	35 ± 6	0.09
Baseline haemoglobin (g/dL)	11.5 ± 2.0	11.5 ± 2.0	11.6 ± 2.0	11.7 ± 2.0	0.006
White blood cell count	10.5 ± 5.5	10.3 ± 5.5	10.6 ± 5.4	10.5 ± 5.3	0.68
Albumin (mg/dl)	3.2 ± 0.5	3.2 ± 0.5	3.2 ± 0.5	3.2 ± 0.5	0.12
BNP (ng/ml)	786 [391–1469]	828 [384–1484]	737 [360–1279]	833 [429–1532]	0.04
Left ventricular ejection fraction (%)	45 ± 18	42 ± 19	42 ± 19	42 ± 19	< 0.0001
**Medications at admission**					
ACEi/ARB	2330 (61)	2066 (61)	1382 (61)	1559 (59)	0.43
Beta blockers	2595 (68)	2239 (66)	1485 (65)	1750 (66)	0.32
MRA	444 (12)	443 (13)	317 (14)	375 (14)	0.006
Loop diuretics	2,889 (75)	2,468 (73)	1,738 (76)	2,070 (78)	0.001
Length of stay (days)	5 [3–9]	5 [3–9]	5 [3–8]	5 [3–9]	0.11

*ACE* angiotensin converting enzyme, *ARB* angiotensin II receptor blockers, *MRA* mineralocorticoid receptor antagonist.

Systolic blood pressure was higher in the winter compared with the summer. Left ventricular ejection fraction of patients admitted in winter was also higher compared to summer. Supplementary Figure 1A demonstrates that the clinical scenario of AHF with elevated blood pressure^15^ was more frequent in winter. Patients admitted in the winter were also more likely to have preserved left ventricular ejection fraction (Supplementary Figure 1B).

### Seasonal variation in admission rates

**Figure 1:**
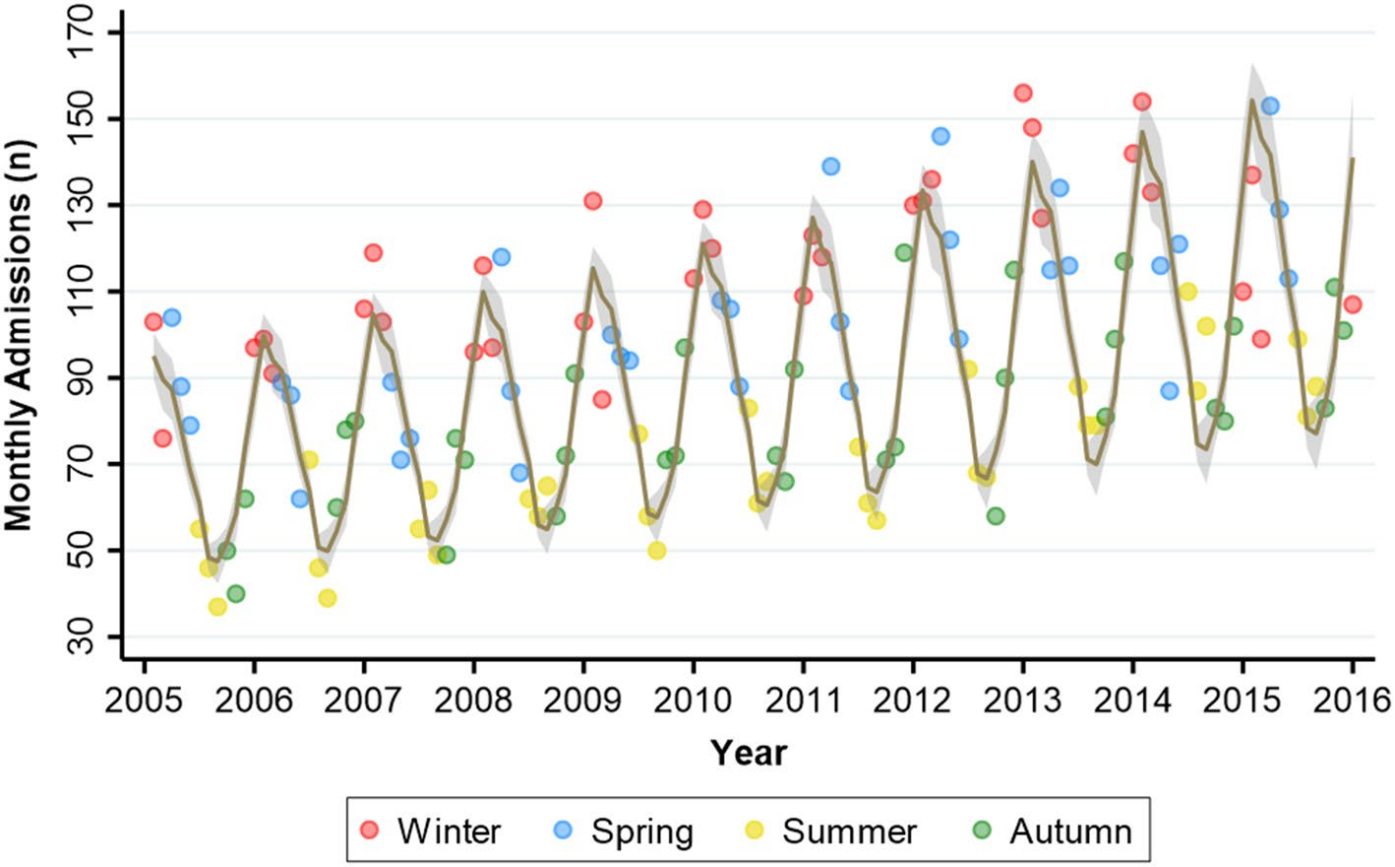
Crude monthly admission numbers from 2005 to 2011. The superimposed curve represents the predicted admissions (with 95% confidence interval) based on a truncated negative binomial regression model.

Figure 1, which plots the monthly AHF hospitalizations over an 11 years period, shows a strong annual periodicity, with grater hospitalizations rates in the winter months compared to summer months (Edwards test χ^2^ = 689.4, *P* < 0.0001). Although the number of monthly admissions increased over time, the annual periodicity was evident in each year (Fig. 1). Periodograms for the same AHF admissions is presented in Supplementary Figure 2 and corresponds to an annual periodicity of disease occurrence. The seasonal variation in AHF admissions was observed in subgroups of patients above and below 75 years and in males and females (Edwards test *P* < 0.0001 for all).

**Figure 2:**
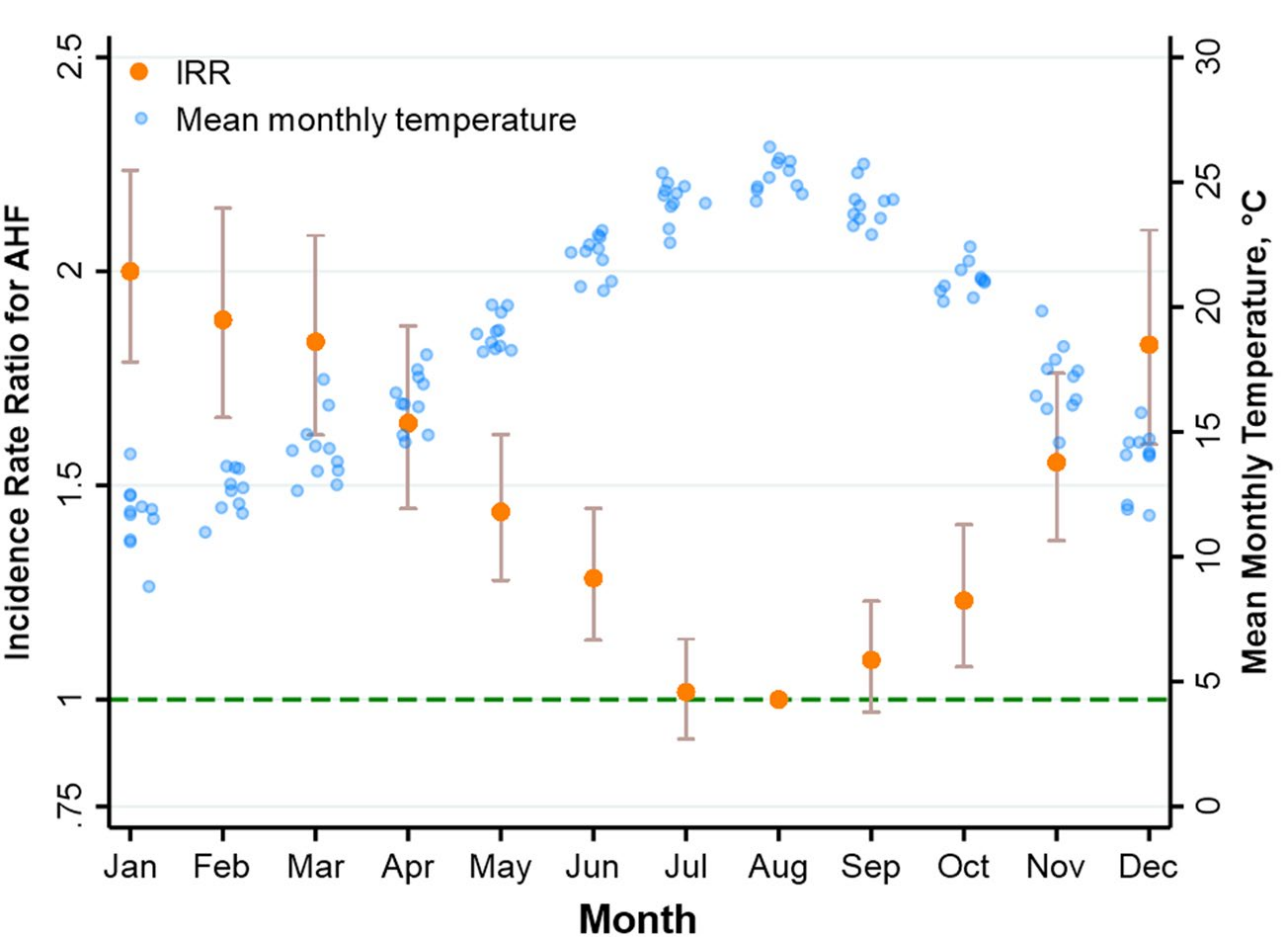
Results of a truncated negative binomial regression for AHF admission over 11 years (2005–2011). The annual pattern of incidence rate ratio (IRR, and 95% CI) is shown (with the reference group being August, the month with the lowest admission rates). The overlaid scatter plot shows the mean monthly temperatures recorded in each month (blue jittered circles) over the 11 years period.

The results of the truncated negative binomial regression model are shown in Fig. 2. The amplitude of the seasonal effect on admission rates (the peak-to-low ratio, January vs. August) was 2.00 ([95% CI 1.79–2.24], *P* < 0.0001).

Figure 2 also shows the close relationship between the mean monthly temperature and the risk of AHF hospitalization, irrespective of the season. There was a strong inverse relationship between the mean monthly admissions and the mean monthly temperature over the 11-year period (IRR 0.95 per 1℃ increase; 95% CI 0.94 to 0.96, *P* < 0.0001). The relationship between mean monthly temperature and the monthly number of admissions was linear, with no apparent threshold (Supplemental Figure 3). Mean monthly humidity was also inversely related to AHF admissions (IRR 0.98 per 1% increase; 95% 0.97 to 0.99, *P* < 0.0001). However, mean monthly humidity was not significantly associated with AHF admission when mean monthly temperature was added to the model.

### Seasonal variation AHF admissions and respiratory infections

**Figure 3:**
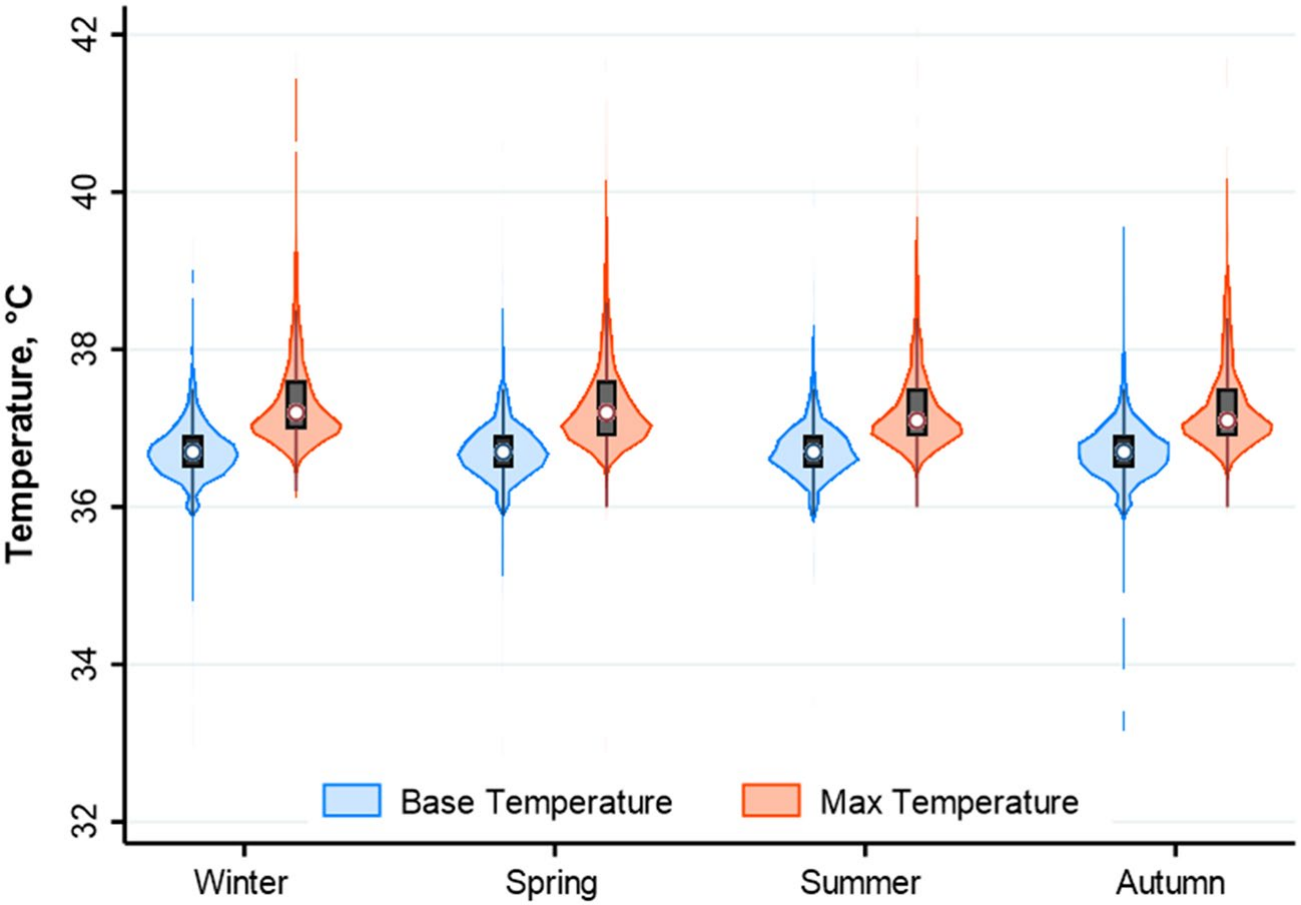
Violin plots of the distribution of baseline temperature and maximal temperature by season. The shape of the violin is the mirrored frequency distribution of the data. Medians are marked by the white dot, and the interquartile range is marked by a vertical black bar, and thin black lines denote the lower and upper range.

Figure 3 displays the distribution of the temperature at admission and the maximal temperature during hospital stay. At admission, fever was present in 1.3%, 1.8%, 1.7% and 2.2% of patients admitted during the winter, spring and summer, and autumn respectively (*P* = 0.30). During hospital course, fever developed in 12.9%, 13.2%, 11.5% and 13.2% of patients admitted during the winter, spring, summer and autumn, respectively (*P* = 0.33). White blood cell counts at admission were similar among the groups (*P* = 0.68).

**Figure 4:**
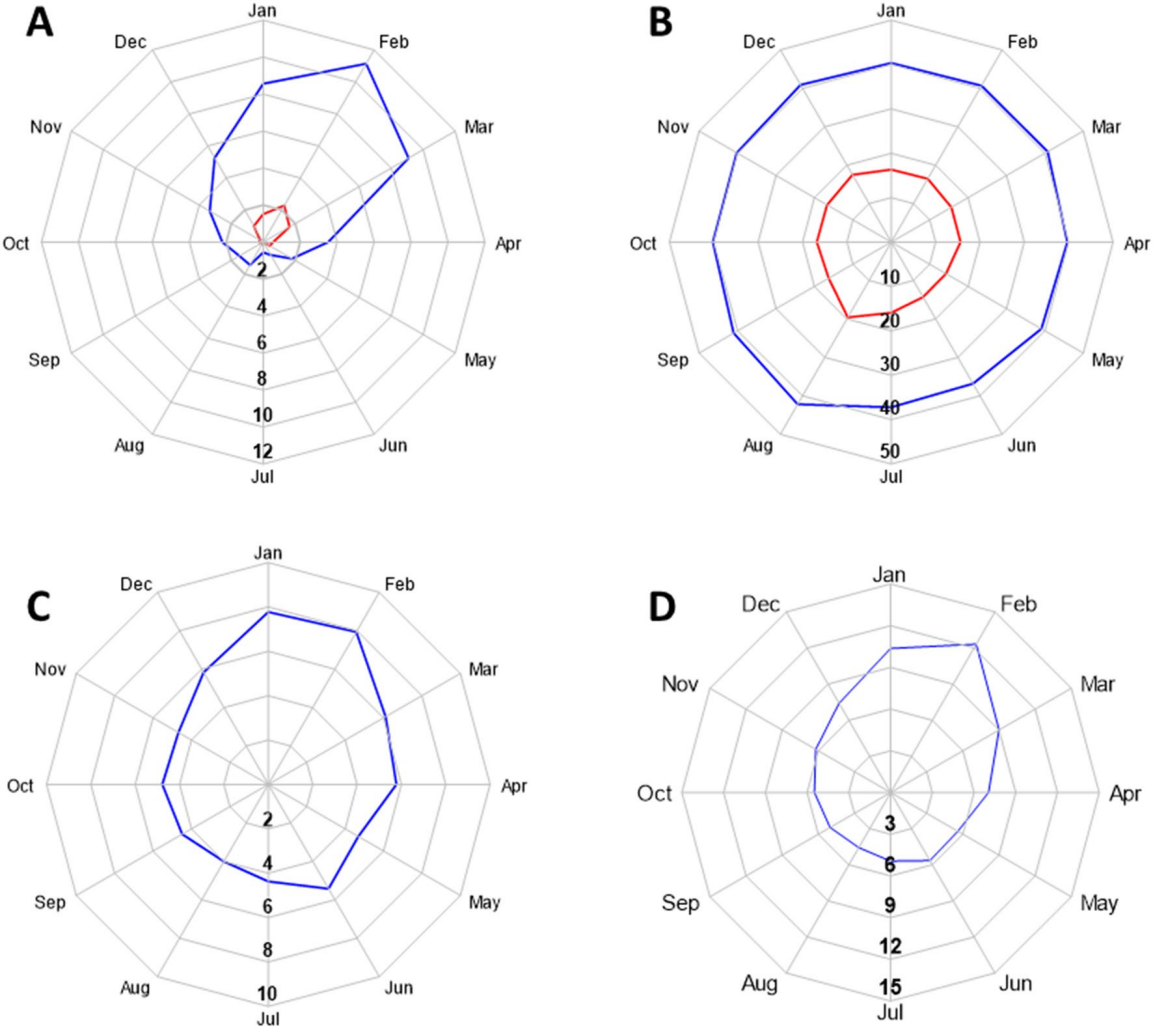
Polar plots of tests and clinical diagnoses performed according to the month of admission. (**A**) Oropharyngeal samples for the influenza virus; (**B**) Bacterial cultures from blood, urine and respiratory secretions; (**C**) Clinical diagnoses of upper respiratory infection, bronchitis or pneumonia; and (**D**) All respiratory infections diagnoses including confirmed influenza infections and influenza-like disease. The blue line represents the percent of cases in each month. The red lines in A and B represent the proportion of positive tests.

Virology tests were obtained in 1,615 patients (13.3%) with strong seasonal variability (Walter & Elwood test χ^2^ = 77.7, *P* < 0.0001; Peak/low ratio 1.91 [95% CI 1.64–2.22]). Tests for influenza were performed in 4.8% of patients, mainly in the winter months, and were positive in 17.1% of tested patients (Fig. 4A). However, overall, only 0.82% of all patient and 1.59% of patients admitted during the winter were positive for the influenza virus.

Blood, urine or respiratory cultures were obtained in 4,947 of patients (41.0%) with small but significant seasonal increase in the summer and autumn (Walter & Elwood test χ^2^ = 6.5, *P* = 0.03; Peak/low ratio 1.22 [95% CI 1.11–1.33]). However, positive bacterial cultures displayed no significant seasonal variation (*P* = 0.49; Fig. 4B).

Clinical diagnoses of upper respiratory infection, bronchitis or pneumonia showed a significant seasonal increase in the winter (Fig. 4C; Walter & Elwood test χ^2^ = 24.2, *P* < 0.0001; Peak/low ratio 1.71 [95% CI 1.36–2.14]). The seasonal pattern of all clinical diagnoses of respiratory infections, confirmed influenza infections, and ILI are shown in Fig. 4D (Walter & Elwood test χ^2^ = 73.3, *P* < 0.0001; Peak/low ratio 2.42 [95% CI 1.394–3.03]).

### Estimation of the Contribution of winter infections to the seasonal variations in AHF admissions

We reanalyzed the seasonality in AHF admission after excluding 898 patients with diagnosis of respiratory infections (including laboratory confirmed influenza) or ILI (42.8%, 27.7%, 12.9% and 16.6% occurring in the winter, autumn, spring and summer, respectively). After exclusion of patients with respiratory infections, the seasonal variation in AHF remained robust (Supplementary Figure 3; Walter & Elwood test χ^2^ = 379.0, *P* < 0.0001; Peak/low ratio January vs. August, 1.81 [95% CI 1.60–2.05]).

### Seasonal changes in clinical outcome

**Figure 5:**
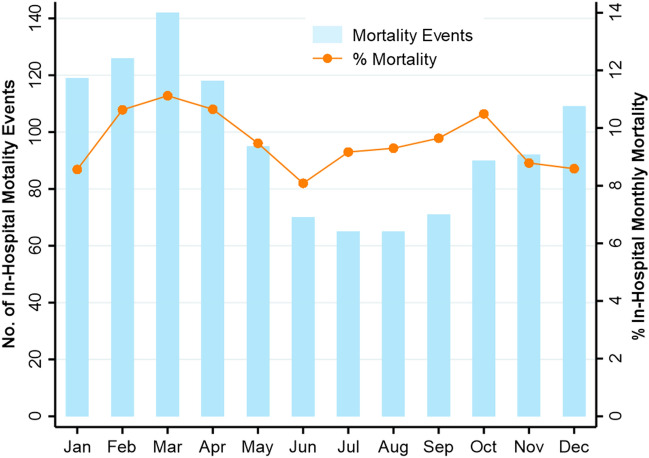
Counts of in-hospital mortality events (blue bars) and the proportion of mortality (orange dots) in each month.

During the 11-years period, the average monthly in-hospital mortality was 9.6%, with higher mortality during winter (bars in Fig. 5). However, accounting for the differences in the population at risk (i.e., higher admission rates during the winter), the proportion of monthly in-hospital mortality (orange line in Fig. 5) showed no seasonal variation (Walter & Elwood test χ^2^ = 1.59, *P* = 0.44).

## Discussion

The current study demonstrated that admissions for AHF followed a striking annual periodicity with winter peak and summer trough. While these finding are consistent with previous studies examining this topic^4,5^, our study adds several novel findings. First, there was no evidence of any clinically meaningful seasonal-based differences in the risk characteristics, indicating that patients admitted in the winter were not “sicker” than those admitted in the summer.

Second, there were no significant differences between patient admitted in different seasons with regard to fever at admission or during hospital stay, or in WBC count. Third, using direct viral tests data, the contribution of the increase in viral diseases during winter (predominantly influenza infections) to AHF admissions was small. Overall, all potential respiratory infections (clinical diagnoses of respiratory infections including ILI and confirmed influenza infections) contributed modestly to the seasonal variation in AHF admissions, accounting for no more than 20% of the seasonal amplitude in AHF infections.

The precise mechanism for the striking seasonal pattern in AHF admissions is not well understood. Several explanations have been invoked to explain the seasonal variations in AHF admissions, including the increase in caloric and sodium intake during winter and increased hemodynamic stress and neurohormonal activation that accompany low ambient temperatures^16^. However, the prevailing hypothesis was that an increase in respiratory infections, especially influenza-related diseases, is the major contributor to the increased AHF winter admissions^4,5^.

Herein we show that the contribution of infectious diseases to AHF oscillations is small. In temperate climates, seasonal influenza arrives in late autumn to early winter, and dissipates in spring^17^. In the current study, this resulted in ~ 2% of winter AHF admissions with laboratory-confirmed influenza infections. Of note, the low prevalence of influenza infection in AHF patient the present study (0.82% of all-seasons admissions) is consistent with a recent report: of 8,189,119 all-cause hospitalizations in patients with HF, only 54,590 (0.67%) had a concomitant discharge diagnosis of influenza infection^18^. Therefore, the prevalence of influenza infection cannot account for the observed magnitude of seasonal AHF changes. After accounting for other respiratory infections, including ILI, the seasonal association of AHF admissions remained robust.

Beyond the prevalence of proven and suspected respiratory infections, other observations about seasonality in the present study are difficult to reconcile with the winter prevalence or virulence of infectious pathogens. Despite expected heterogeneity in the frequency and severity of influenza infections, there were no deviations from the simple annual pattern over 11 years, irrespective of the strong trend of increasing AHF admissions. This suggests the presence of environmental factors that affect the susceptibility of the population at risk and synchronizes seasonal variation. Indeed, beyond winter respiratory infections, we observed a close relationship between monthly temperature fluctuations and AHF admissions that was consistent throughout the years. For example, AHF admissions were substantially lower in the hotter autumn compared with the spring, despite similar rates of respiratory infections during these periods.

Cold and heat exposure provokes both acute and chronic physiological changes modulated by air temperature and humidity, that have cardiovascular consequences relevant to heart failure. The predominant thermoregulatory response to a fall in peripheral temperatures and a potential fall in core temperature is peripheral vasoconstriction to reduce thermal conduction via the skin. Largely mediated via sympathetic activation, these responses also result in an elevated heart rate and blood pressure^19–21^, and are accompanied by increased metabolic demand to maintain relatively higher resting metabolic rates^20^. By contrast, exposure to elevated temperatures provokes generalized peripheral vasodilatation and sweating, which can lead to substantial fluid and salt losses^22^.

In this context, in addition to winter period representing a trigger for AHF, hot weather may afford some protection from heart failure decompensation. Rehospitalizations for HF typically entail a gradual fluid and sodium accumulation leading to weight gain and a rise in ventricular filling pressures that begin weeks prior to overt clinical symptoms^23–25^. Hence, higher active sweating in the summer^26^, often culminating in 500–700 mL daily^27^, may mitigate the ongoing fluid accumulation that precedes decompensation. Such mechanism may predominate in a hot climate (e.g., Mediterranean) comprising mild winters and hot summer months. Importantly, because these seasonal changes affect a large proportion of the population, they may be sufficient to create large seasonal surges in disease incidence.

Mortality was higher in patients admitted during the winter, but this was solely owing to the higher admissions rate during this period, implying similar individual mortality risk during all seasons. Increased mortality in AHF in the winter may be expected if these patients represent a more susceptible population, or if the seasonal trigger for hospitalization is severe enough to increase the mortality risk. In the current study, the similar in hospital mortality rates is a likely finding given the similarity in the baseline characteristics.

### Study limitations

It is important to consider several limitations pertaining to the methods of this study. First, this was a single-center retrospective analysis, and thus, the results must be regarded as hypothesis-generating and exploratory and require validation in other studies. The infectious disease surveillance may not capture all respiratory cases due to underreporting or underscreening, potentially leading to underestimation of their effects. However, not all concomitant respiratory infections can be considered as the primary drivers for hospitalization. The magnitude of the seasonal amplitude and the overall effect of ambient temperatures on AHF risk may differ in other parts of the world.

It is important to emphasize that excess winter AHF event may not be a good indicator of the overall effect of respiratory infections as a significant proportion of those occur outside of this period, presumably due to the higher susceptibility of heart failure patients to these infections and their complications.

## Conclusion

These is a dominant seasonal modulation of AHF admissions which is only partly explained the incidence of winter respiratory infections. There is a strong inverse relationship between temperature and AHF admissions, indicating that environmental factors modify the susceptibility of HF patients to decompensation. A decrease in susceptibility of the HF population during high temperature periods, perhaps linked to peripheral vasodilatation and the increased fluid and salt loss via perspiration, might account for annual troughs in clinical decompensation. More data is needed with regard to the pathophysiologic mechanisms underlying seasonal changes in the susceptibility of HF patients to decompensation.

## Supplementary Information

Supplementary Information.
