# Supplementary material for: Environmental factors, winter respiratory infections and the seasonal variation in heart failure admissions
Source: Sci Rep. 2021 May 28;11:11292. doi: 10.1038/s41598-021-90790-7 (PMC8163784; doi:10.1038/s41598-021-90790-7)
Supplement: Supplementary file 1 — Supplementary Information. [file 41598_2021_90790_MOESM1_ESM.docx]

Environmental Factors, Winter Respiratory Infections and the Seasonal Variation in Heart Failure Admissions

### Doron Aronson MD

### Supplementary material


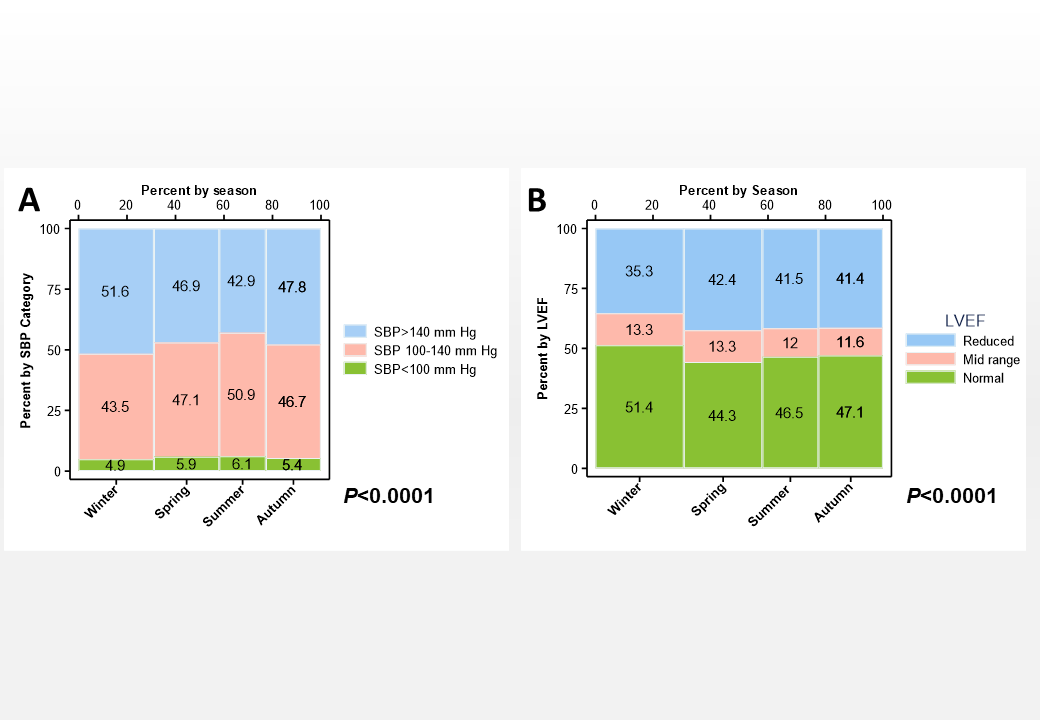


**Supplementary Figure 1:** **(A)** Systolic blood pressure at admission based on the clinical scenario classification of AHF proposed by Mebazaa et al. (Crit Care Med 2008;36: S129-39). Initial systolic blood pressure (SBP) is classified as SBP ≥140mmHg; 100≤SBP<140mmHg; and SBP <100mmHg. **(B)** Patients admitted in the winter were also more likely to have normal left ventricular ejection fraction.

**Supplementary Figure 2:** The spectrum (defined as the maximum squared amplitude of a cosine regression model with a given frequency) is plotted against underlying frequency. The largest peak is seen at a frequency of 1/12, corresponds to annual periodicity of disease occurrence.

**Supplementary Figure 3:**  Exposure–response relationship between mean monthly temperature and admissions for AHF.

**Supplementary Figure 4:** Results of a truncated negative binomial regression for AHF admission over 11 years (2005-2011) after exclusion of patients with respiratory infections. The annual pattern of incidence rate ratio (and 95% CI) is shown (with the reference group being August, the month with the lowest admission rates). The overlaid scatter plot shows the mean monthly temperatures recorded in each month (blue jittered circles) over the 11 years period.
